# The pH-Dependent Specificity of Cathepsin S and Its Implications for Inflammatory Communications and Disease

**DOI:** 10.1021/acs.biochem.5c00287

**Published:** 2025-07-19

**Authors:** Riley DeHority, Laura I. Gil Pineda, Kari Cochran, Bentley Chen, Daniel Bratek, Richard F. Helm, Justin A. Lemkul, Chenming Zhang

**Affiliations:** † Department of Biological Systems Engineering, 1757 Virginia Polytechnic Institute and State University, Blacksburg, Virginia 24061, United States; ‡ Department of Biochemistry, Virginia Polytechnic Institute and State University, Blacksburg, Virginia 24061, United States

## Abstract

Proteases have two major roles in health and disease: making functional changes to proteins as a post-translational modification and degradation of proteins as a regulatory or waste management mechanism. The cysteine protease cathepsin S serves both of these functions. It digests antigens in the adaptive immune system and is associated with many autoimmune diseases and cancers. Here, we show that the catalytic specificity of human cathepsin S is regulated by the pH conditions of its environment and identify the structural determinants of this switch. Peptide digests show that the proteolytic specificity of cathepsin S narrows at extracellular pH. Crystal structures reveal that a lysine residue descends into the S3 pocket of the active site above pH 7, which can be explained by changes in the protein's surface charge at that pH. We discuss biological compartment transitions and disease processes associated with cathepsin S in which these pH-dependent specificity switches may be triggered.

## Graphical abstract

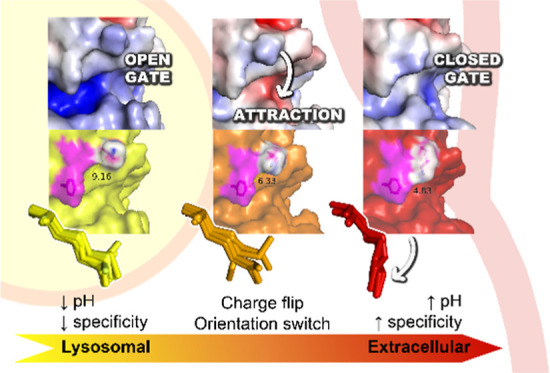

## Introduction

Cathepsin S is a cysteine protease that is critical for the adaptive immune response, and its dysregulation is linked to the pathogenesis of dozens of major human diseases. It plays a role, either pathological or protective, in each of the top ten global causes of death (Table 1).^1^ In the adaptive immune system, it digests antigens in the endolysosomal pathway and prepares the MHC-II receptor for antigen loading.^2–4^ While it is only constitutively expressed at high levels in the spleen and in professional antigen presenting cells,^5^ it can be upregulated by cytokines like interferon-γ ^4,6,7^ and secreted into the extracellular environment by macrophages,^8–10^ endothelial cells,^11^ or smooth muscle^12^ cells. It remodels the extracellular matrix during the course of autoimmune diseases like atherosclerosis,^13–16,68^ rheumatoid arthritis,^11,17–19^ multiple sclerosis,^20–22^ Sjögren’s syndrome,^23,24^ psoriasis,^25^ and periodontitis.^26^ It contributes to the structural damage of lupus: in fact, one popular lupus mouse model works in part by overexpressing cathepsin S.^27–29^ It evokes the sensation of itch,^30^ promotes the progression of cerebral aneurysms,^31^ may be a major cause of idiopathic nerve pain,^32,33^ and even contributes to the elastolytic process of skin aging.^34^ Cathepsin S dysregulation is implicated in many cancers: it promotes neovascularization and tumor growth,^35–39^ and in cathepsin S knockout mouse models, metastatic breast cancer cells become unable to cross the blood–brain barrier.^40^ For a protein whose expression is highly regulated and tissue specific, cathepsin S has ubiquitous connections to the world’s most devastating diseases. Beyond its inflammatory roles, it is associated with cell adhesion, regulates calcium homeostasis,^41^ and activates sodium channels.^42^

**Table 1:** The World Health Organization’s Most Recent Ranking of the Top 10 Global Causes of Death^1^ and Their Association with Cathepsin S

rank	disease	cathepsin S association
1	ischemic heart disease	• activates the protease activated receptor 2 (PAR-2) during atherosclerotic plaque formation
		• digests the elastin of vasculature during plaque expansion
		• plays a role in plaque destabilization and rupture^15^
		• gene is highly expressed during all stages of atherosclerosis^14,43^
		• elevated both before and after ST-segment elevation myocardial infarctions (STEMI)^44^
		• contributes to myocardial ischemia/reperfusion (I/R) injury^45^
		• critical for revascularization during recovery^46,47^
2	COVID-19	infectious diseases generally
		• processes antigens before MHC loading^4,48,49^
		• critical for the liberation of the invariant chain from the MHC–II complex facilitating antigen presentation^3,49,50^
		COVID-19 specifically
		• cleaves the spike protein in multiple locations^51^
3	stroke	• highly expressed after strokes^52^
		• increases neuroinflammation and blood–brain barrier leakage^52^
		• associated with cerebral infarctions during strokes^53^
		• critical for revascularization during recovery^46,47^
4	chronic obstructive pulmonary disease (COPD)	• cigarette smoking leads to the upregulation of cathepsin S in the lungs^54,55^
		• associated with the degradation of lung epithelial cells in COPD^56,57^
5	lower respiratory infections	general role in antigen processing and presentation, plus
		• contributes to lung inflammation and degradation of both structural and antimicrobial lung proteins in chronic lung infections, especially in cystic fibrosis^58–60^
6	trachea, bronchus, lung cancers	• elevated levels are associated with many types of cancer, and in many of those types, higher levels are associated with worse patient outcomes^35^
		• lung cancer patients with low levels of cathepsin S in tumors exhibit significantly higher risk of death^61^
		• inhibition reduces lung cancer cell migration^62^
		• lung cancers do not grow and vascularize as quickly without cathepsin S^64^
7	Alzheimer’s disease and other dementias	• found at increased levels in the post-mortem brains of patients with Alzheimer’s disease^64,65^
		• processes the amyloid precursor protein found in neuritic plaques^64^
		• cleaves tau and leads to tau oligomer aggregation^66^
8	diabetes mellitus	• increased levels associated with diabetes^67–69^
		• activated PAR-2 induces vascular injury in diabetes^70^
9	kidney diseases	• levels increase with the progression of chronic kidney disease, along with aortic stiffening^71^
		• associated with glomerular filtration rate decline^72^
		• inhibition lowers kidney damage in lupus nephritis models^73^
		• inhibition lowers atherosclerosis in chronic renal disease models^74^
10	tuberculosis	general role in antigen processing and presentation, plus
		• tuberculosis produces a microRNA, which binds to cathepsin S, RNA leading to downregulation during infection and a lowered immune response^75,76^

Cysteine proteases break down proteins by hydrolyzing a target peptide bond. Their catalytic specificity, meaning the amino acid sequences that they cleave most readily and the speeds at which those reactions happen, is determined by the shape and physicochemical properties of their active sites. The catalytic triad of cathepsin S is Cys-25, His-164, and Asn-184 (papain numbering), where cysteine and histidine act as a proton donor–acceptor pair. The catalytic specificity of cathepsin S is still not well understood despite four decades of characterization experiments.^49,77–83^ It is well established that cathepsin S cleaves peptide sequences near hydrophobic residues, specifically with hydrophobic residues in the P2 position^84^ (the second amino acid away from the cleavage site in the C-terminal direction) as cathepsin S has a deep hydrophobic pocket (S2) to accommodate it. This promiscuous specificity allows cathepsin S to thoroughly degrade whole proteins in its role as a lysosomal protease. However, it is also secreted into the extracellular environment to play cell signaling and receptor activation roles, often cleaving specific signaling domains from larger proteins: an unlikely pattern of activity for a promiscuous protease.^40,82,85^ Cathepsin S remains active in neutral environments, with an active pH range of 4–8.5.^77,86,87^ This property makes it unique: other lysosomal proteases are rapidly inactivated by neutral or basic pH,^88^ which protects the extracellular environment from rogue proteolysis by secreted proteins. Given its broad active pH range and broad substrate specificity, one would expect cathepsin S to promiscuously degrade structural proteins outside of the cell, which it is known to do in the context of autoimmune diseases, limited only by its natural inhibitors like cystatin C.^20,86,89^ However, instead of the expected general destruction, efforts to replicate these extracellular environments *in vitro* show specific and limited cleavage of proteins by cathepsin S that can often easily be blocked by one or two amino acid substitutions in the substrate.^42,82,90–92^

We hypothesized that pH regulates the specificity of cathepsin S, not by inactivating it but by limiting its substrate specificity at extracellular pH, allowing for nonspecific degradation in the lysosome and controlled release of signaling molecules in the extracellular environment. Studies of cathepsin S digestion of whole proteins at a variety of pH levels^40,82,93,94^ identified different patterns of degradation between lysosomal, endosomal, and extracellular pH; however our comprehensive review of those studies did not reveal obvious patterns (data not published). We set out to characterize this varying specificity of cathepsin S and identify its structural determinants.

Other cysteine proteases, including legumain, cruzain, and cathepsin B, have been identified as having pH-dependent specificities. Legumain switches from an endopeptidase, to a carboxypeptidase, to a ligase based on the pH of its current environment as well as the environment it was activated in.^81,95,96^ A pocket on the active site of cruzain changes shape based on whether a histidine residue is protonated and accepts hydrophobic residues in that pocket at a wider pH range than it accepts basic residues.^97^ In the case of cathepsin B, the protonation state of two histidine residues controls a loop of the protein that gates off part of the active site at low pH, allowing only exopeptidase activities. At neutral pH, the gate is opened and cathepsin B can act as an endopeptidase as longer substrates can fit into its binding cleft.^98,99^ This conformational change has allowed pH-specific inhibitors to be developed for cathepsin B, limiting inhibition to compartments of interest.^100,101^

Identifying the structural determinants of cathepsin S specificity may allow for the development of compartment-specific inhibitors. While cathepsin S has been identified as a therapeutic target for autoimmune diseases and cancers, efforts to treat these diseases with cathepsin S inhibitors have not been successful.^102–104^ If the structure of cathepsin S changes with the environment, inhibitors could be developed that are targeted more precisely to the form of cathepsin S in the compartments where it is contributing to pathology and damage. This would also minimize an inhibitor’s effect on healthy cathepsin S signaling pathways. For example, cathepsin S inhibitor RO5459072 reduces B cell counts. In two clinical trials, itchiness was reported as an adverse reaction to the drug.^105,106^ There are many pathways to the itch sensation, but cathepsin S causes the itch sensation by cleaving specific extracellular regions of the G-protein-coupled receptors PAR-2, PAR-4, and MrgprC11.^30,107,108^ It may be that the inhibitors used in these trials were effective only against the structure of cathepsin S found in lysosomal conditions. An inhibitor specific to lysosomal cathepsin S may not impact extracellular activities, allowing any cathepsin S-associated itch sensation to continue unabated. Alternatively, an inhibitor specific to the extracellular environment might halt the contribution of cathepsin S to itch but might allow lysosomal activities such as antigen processing to continue. Beyond building more precise inhibitors, a more complete characterization of the biochemical regulation of cathepsin S is critical for the vaccine engineer, the oncologist, and the public health researcher who wish to predict how a particular antigen will be processed and eventually bring about immunological memory.

## Methods

### Experimental Peptide Selection

A literature review showed that the P3–P2′ substrate positions were generally accepted as playing some role in cathepsin S specificity, and P4–P6′ had at least one catalysis study, which suggested those positions were selective; therefore, 10-amino acid long peptides from P4–P6′ were designed. Sequences from the cathepsin S specificity literature were collected and screened in a methods review to identify peptides predicted to occupy the extremes of pH-dependent binding patterns. Missing residues were replaced by residues from the invariant chain peptide LPMGALPQGP.^49^ Six peptides (IGPGGVAAAA, LPMGALPQGP, HRVKALPQGP, LIFQQGHPDH, LIFEQGHPDH, HVVQLFIQGP, and LRDRPRMMRR, predicted to be a negative control) were initially chosen for comparison (see Supporting Information—Peptide Selection).

### Peptide Digests

Initial peptide digests were performed at a 55 nM concentration of human cathepsin S (R&D Systems, Minneapolis, MN), 1 mM concentration of the peptides (LifeTein, Somerset, NJ), and 6 mM concentration of dithiothreitol (DTT, Sigma-Aldrich, St. Louis, MO) in a 0.1 M sodium acetate/acetic acid buffer (pH 5) or 1× PBS (pH 7.4) for a total reaction volume of 400 μL. Buffers and DTT were filter-sterilized with a 0.22 μM PVDF syringe filter (CellTreat, Pepperell, MA). Peptides were dissolved in either nuclease-free water or 70% isopropanol depending on net charge. Cathepsin S stock solution was diluted to 2 μM in nuclease-free water and allowed to come to room temperature before use. Aliquots of 100 μL were taken at time point zero (before the addition of cathepsin S), 15 min, 30 min, and 16 h.

To check that the pH effects seen in the screening experiment were due to pH and not other ionic impacts, all future reactions used isotonic citrate-phosphate buffers for all pH values, with 0.3 M citric acid (BioWORLD, Columbus, OH) titrated into 0.1 M disodium phosphate (Fisher Chemical, Pittsburgh, PA) (see Supporting Information—Buffers). The pH range digest of peptides HRVK and LIFQ, along with a third peptide LVVR|ALPQGP, occurred at pH 4.5, 5.0, 5.5, 6.0, 6.5, 7.0, 7.2, 7.4, and 7.6 (±0.01) measured using a calibrated Accumet AR-15 pH meter (Fisher Scientific, Pittsburgh, PA). Peptides were dissolved in 2.4% isopropanol v/v, a concentration that did not impact the titration curve of the buffer system. Frozen activated cathepsin S stock solution was diluted to 2.5 μM in nuclease-free water and allowed to warm to room temperature before use. Results for peptide LVVR|ALPQGP were similar to those for LIFQ, except for increased uncertainty between pH 5.0 and 6.0 (Supporting Information—LVVR pH Curve).

There were two reactions per pH level per peptide, which included 300 μM peptide, 4 mM DTT, and 30 nM cathepsin S. Each set of replicates was paired with an undigested reference sample at pH 6.5 with 4 mM DTT and no enzyme. All samples were heat inactivated after 30 min, and the percent digestion was calculated by comparing substrate peak areas of each set of digest samples with their paired undigested reference peak.

Other digestions were performed using ammonium acetate, triethylammonium acetate, and triethylammonium bicarbonate (data not shown). Triethylammonium bicarbonate (TEAB) was found to block most of the cathepsin S activity.

All reactions were prepared in a biological safety cabinet, were incubated at 37 °C, and were stopped by heat denaturation at 90 °C for 5 min in a water bath.

### LCMS and Analysis of Data

Analyses were performed on a Shimadzu LCMS9030 QToF interfaced with a LC-40B X3 UPLC, a SIL-40 C X3 autosampler (10 °C), and a CTO-40 C column oven (40 °C). Gradient separations utilized a BEH C_18_ column (2.1 mm × 50 mm, 1.7 μm particle size; Waters). Solvent A (0.1% formic acid in water) and solvent B (0.1% formic acid in acetonitrile) were at a constant flow rate of 0.4 mL/min. Initial conditions were 95:5 (A:B), which was held constant for 2 min, followed by a shallow linear gradient to 30% B at 7 min, then to 95% B at 9 min, which was held for 2 min. The gradient was converted to starting conditions with a 1 min gradient to 5% B, followed by a 3 min hold. Sample injection volumes ranged from 2 to 5 μL. The first minute of the separation was diverted to waste to avoid contamination of the mass spectrometer interface with buffer salts.

The mass spectrometer was operated in positive ion mode using electrospray ionization (150–1500 *m*/*z*) and external calibration (NaI). Interface voltage was 4.0 kV at 300 °C, with a desolvation temperature of 526 °C and a DL transfer line temperature of 250 °C. Gas flows (l min^–1^) were 2, 10, and 10 for nebulizing, heating, and drying gases, respectively.

LCMS results were analyzed using Shimadzu LabSolutions Browser Version 5.118. Peak areas were calculated by using the i-PeakFinder algorithm and the vertical division baseline type. Peaks were identified using their spectra and comparing it to expected spectra using UCSF’s Protein Prospector tools.^141^

### S3 Pocket Visualizations and Distance Measurements

Lys-64 orientations were inspected in all available PDB structures of cathepsin S. For the visualization in Figure 3, all WT structures at all pH levels were included, with the exception of PDB 9GJ2 chain B, which failed multiple attempts at alignment but followed the same pattern as the other pH 4.6 structures. PDB structures were aligned using PyMOL’s Align tool at 10 steps. S3 pocket distances for all WT crystal structures and all pH 4.5 C25S structures available in the PDB as of October 2024 were measured using PyMOL’s Measure tool to two decimal places measuring from the terminal amine of Lys-64 to the ζ carbon of Phe-70. All 3D structure visualizations and measurements were done in PyMOL version 2.5.2.

### Electrostatic Fields and Analysis

For electrostatics visualization, PDB chains were isolated and the PDB2PQR web server was used to protonate them to their pH of crystallization using the included PROPKA and the PARSE force field.^142^ Then, the PyMOL APBS plugin (PyMOL version 2.5.2) was used to generate electrostatic surfaces for all structures at pH 4.2, 4.56, 4.8, 6.5, 7, and 7.3 and the same five representative structures from 4.6 and 5.0.

### Statistics

Statistics were performed in GraphPad Prism version 10.3.1. Substrate–product curves were calculated by using a simple linear regression. Comparisons between inhibited and uninhibited S3 pocket widths at pH 4.5 and 5.0 were calculated using unpaired *t-*tests. A linear regression with S3 pocket distances as an outcome and with input variables pH of crystallization and presence of an inhibitor in the S3 pocket (binary) were analyzed using a multiple linear regression analysis. The interaction effect between pH and inhibitor presence was insignificant and therefore was removed from the model. All of these were tested for normality by using quantile–quantile plots.

## Results

### Cathepsin S Degrades More Substrates at Lysosomal pH

We screened the literature for cathepsin S substrates and identified six peptides (Figure 1A)^34,49,78,80^ to digest in a screening experiment, taking samples of the reaction over 16 h (Figure 1B). This screening, at lysosomal pH 5.0 (sodium acetate) and extracellular pH 7.4 (PBS), revealed three patterns of activity: rapid digestion at similar rates between the two pH levels for more basic peptides (Figure 1D), slower digestion overall plus more complete digestion at pH 5.0 than 7.4 for more neutral and hydrophobic peptides (Figure 1E), and one peptide that was not digested at all at pH 5.0 but was partially digested at pH 7.4 (Figure 1C). A pattern of narrowed specificity at increased pH had been previously noted in the digestion of thyroglobulin^82^ and junctional adhesion molecule^40^ proteins. Higher cleavage levels at higher pH as seen with peptide LIFE|QGHPDH (henceforth “LIFE”, with | representing the cleavage site) is an uncommon finding, but has been seen previously in the digestion of bovine laminin.^94^ Intriguingly, rather than the clear binary, we expected to see with some substrates accepted at the higher pH and all substrates accepted at the lysosomal pH; the results show varying amounts of each substrate digested between the two pH levels, demonstrating a complex relationship between pH and activity.

**Figure 1:**
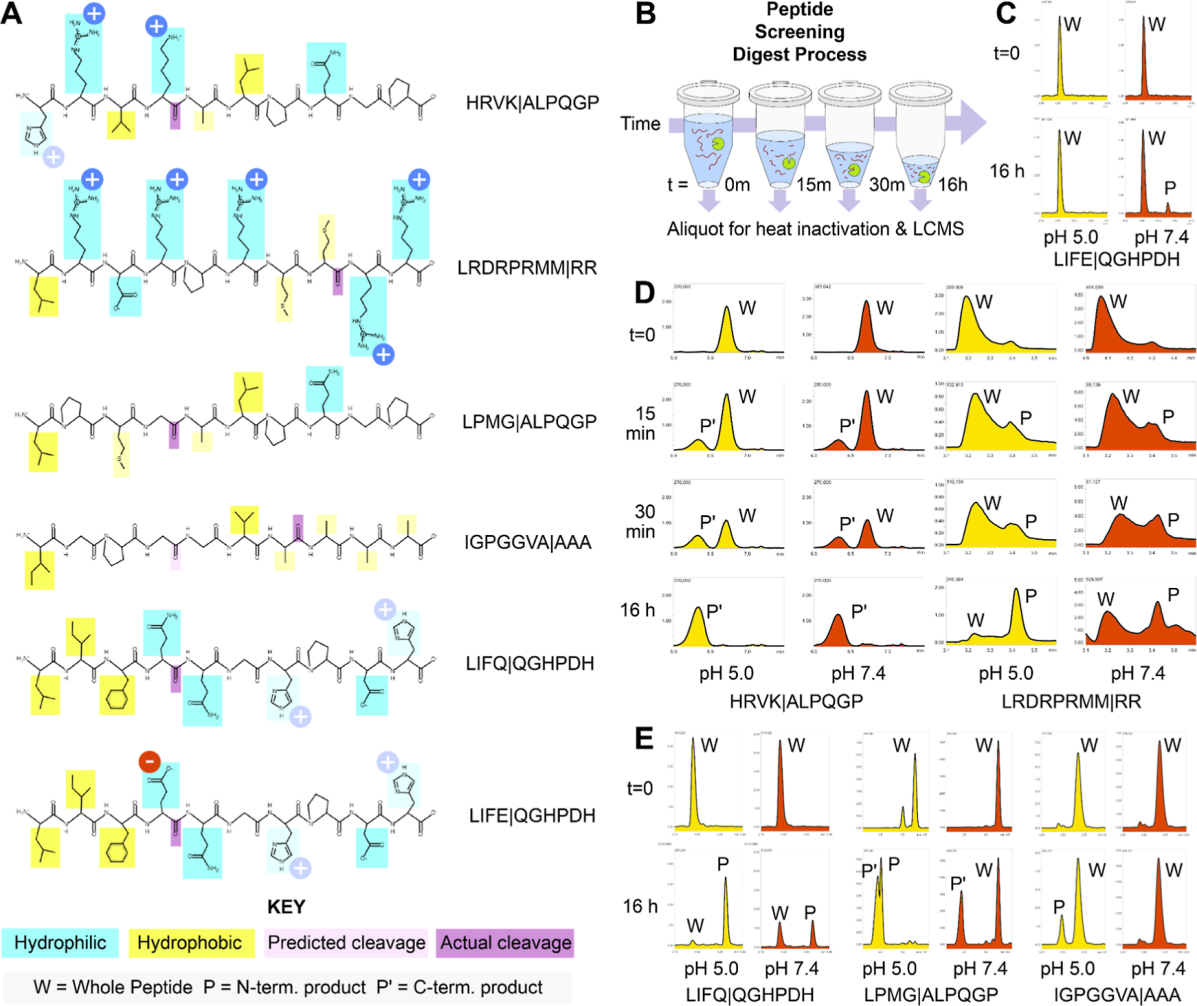
Peptide structures and results of the initial pH screening digest. (A) Synthesized peptide structures labeled with hydrophobic and hydrophilic residues, charges (with histidine shown as light blue, as they are likely deprotonated between the pH values of interest), and cleavage locations, both expected and actual. Drawn with PEP Draw. (B) Diagram of the screening digest experiment, with aliquots removed at *t* = 0, 15 min, 30 min, and 16 h. (C) LCMS graphs (abundance v. retention time) for peptide LIFE|QGHPDH, which showed digestion at pH 7.4 only, with pH 5.0 samples in yellow and pH 7.4 samples in red. (D) LCMS graphs for peptides, which showed digestion within 15 min. (E) LCMS graphs for peptides, for which digestion was more complete at pH 5.0.

### A Specificity Flip Occurs around pH 7.2

Two of these peptides (LIFQ|QGHPDH—henceforth “LIFQ” and HRVK|ALPQGP—henceforth “HRVK”) were selected for further study and were digested across a pH range from 4.5 to 7.6 (citrate-phosphate buffer) for 30 min. This approach revealed two distinct patterns of cathepsin S catalysis (Figure 2A). Both pH curves peak at pH 6.5, which aligns with the established pH optimum of cathepsin S.^77^ However, between pH 6.5 and 7.2, a divergence occurs: peptide LIFQ continues to be readily digested, but digestion of peptide HRVK decreases as pH increases. This is clear evidence of a cathepsin S specificity switch of in a pH environment that mimics the endosomal-extracellular boundary. Intriguingly, while peptide LIFQ continued to be digested readily between pH 6.5 and 7.6 (Figure 2B), its product decreased above pH 7.2 (Figure 2C). While there is a clear linear relationship between substrate peak disappearance and product peak appearance for all pH levels of HRVK digestion (Figure 2D), above pH 7.2, the product peak areas for LIFQ digestion all fall below a regression line (Figure 2E). While a secondary product could not be identified to explain the discrepancy between expected and actual product area based on substrate disappearance, it is clear that the digestion of LIFQ also underwent a specificity switch around 7.2, with either further degradation of the product that did not occur at lower pH values or a certain percentage of catalysis switching to a different cleavage location on the original peptide substrate. Therefore, unexpectedly, the digestion of both peptides points to a pH-dependent specificity switch for cathepsin S around pH 7.2.

**Figure 2:**
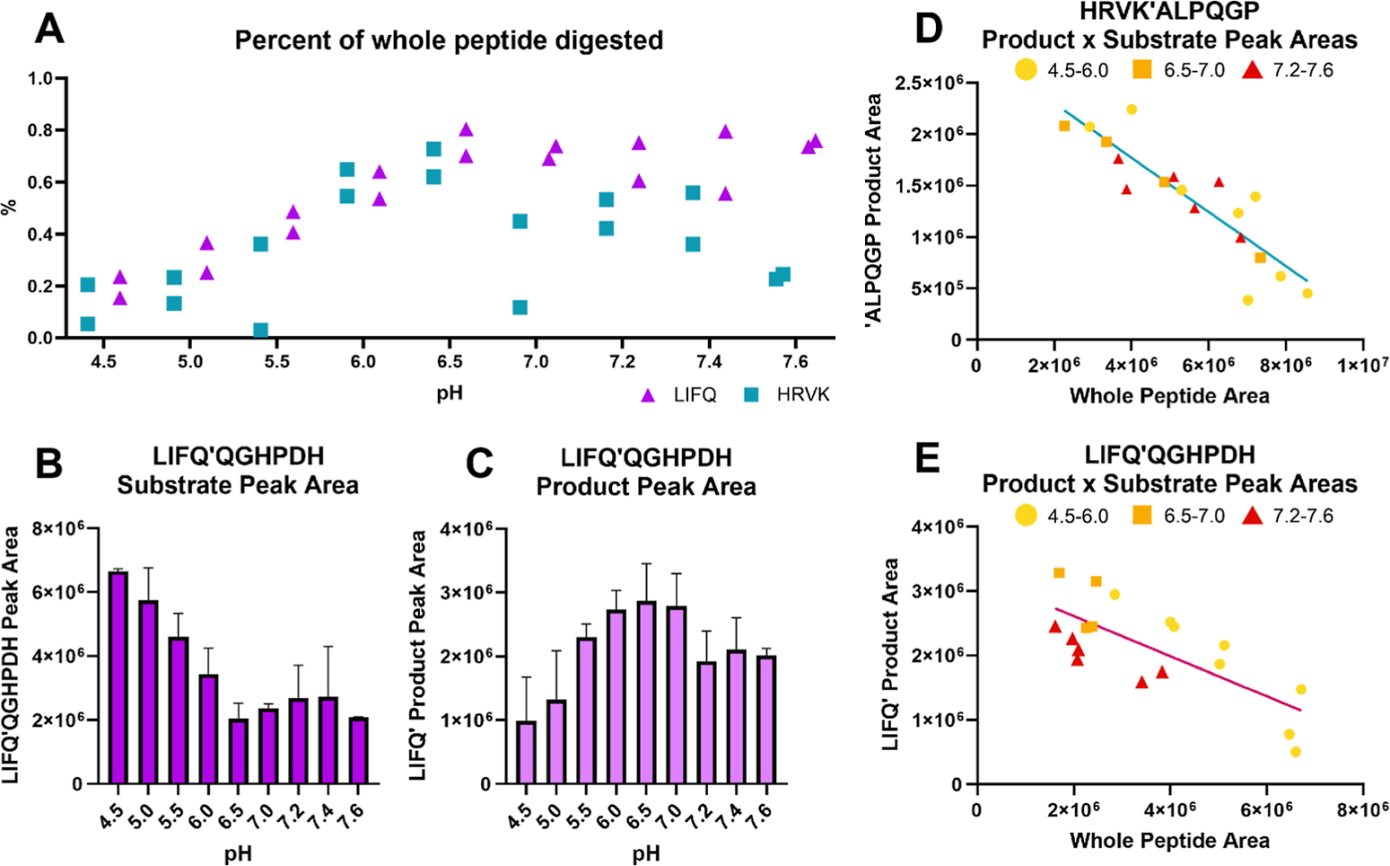
Results for the digestion of peptides HRVK and LIFQ over the pH range 4.5–7.6. (A) Percent of the peptide substrate digested, calculated as the substrate peak after 30 min compared to an undigested reference peak (*n* = 2). (B) Peptide LIFQ substrate peak area over pH. (C) LIFQ product peak area graph, with an unexpected dip at pH 7.2 and above. (D) Peak areas for the product peak area vs the substrate peak areas for each individual sample from peptide HRVK, with pH groups represented in color and with a simple linear regression line shown (*p* < 0.0001, *R* ^2^ = 77.65%). (E) Peak areas for the product peak area vs the substrate peak areas for peptide LIFQ, with a simple linear regression line shown (*p* = 0.0004, *R* ^2^ = 55.30%). All samples at and above pH 7.2 sit below the LIFQ regression line.

### Electrostatic Potential Calculations Reveal Active Site Potential Flip between 7.0 and 7.4

To identify structural determinants for this specificity switch, all publicly available cathepsin S crystal structures were compared, including 28 Protein Data Bank (PDB) entries making up 57 individual wild-type (WT) crystal structures of cathepsin S for which the pH of crystallization was available. By comparing these 57 crystal structures, we found that the S3 pocket of the active site, made up of residues Gly-62, Lys-64, and Phe-70, showed a consistent pH-dependent trend across all available structures, with Lys-64 descending into the active site as pH increases, closing the S3 pocket (Figure 3A).

**Figure 3:**
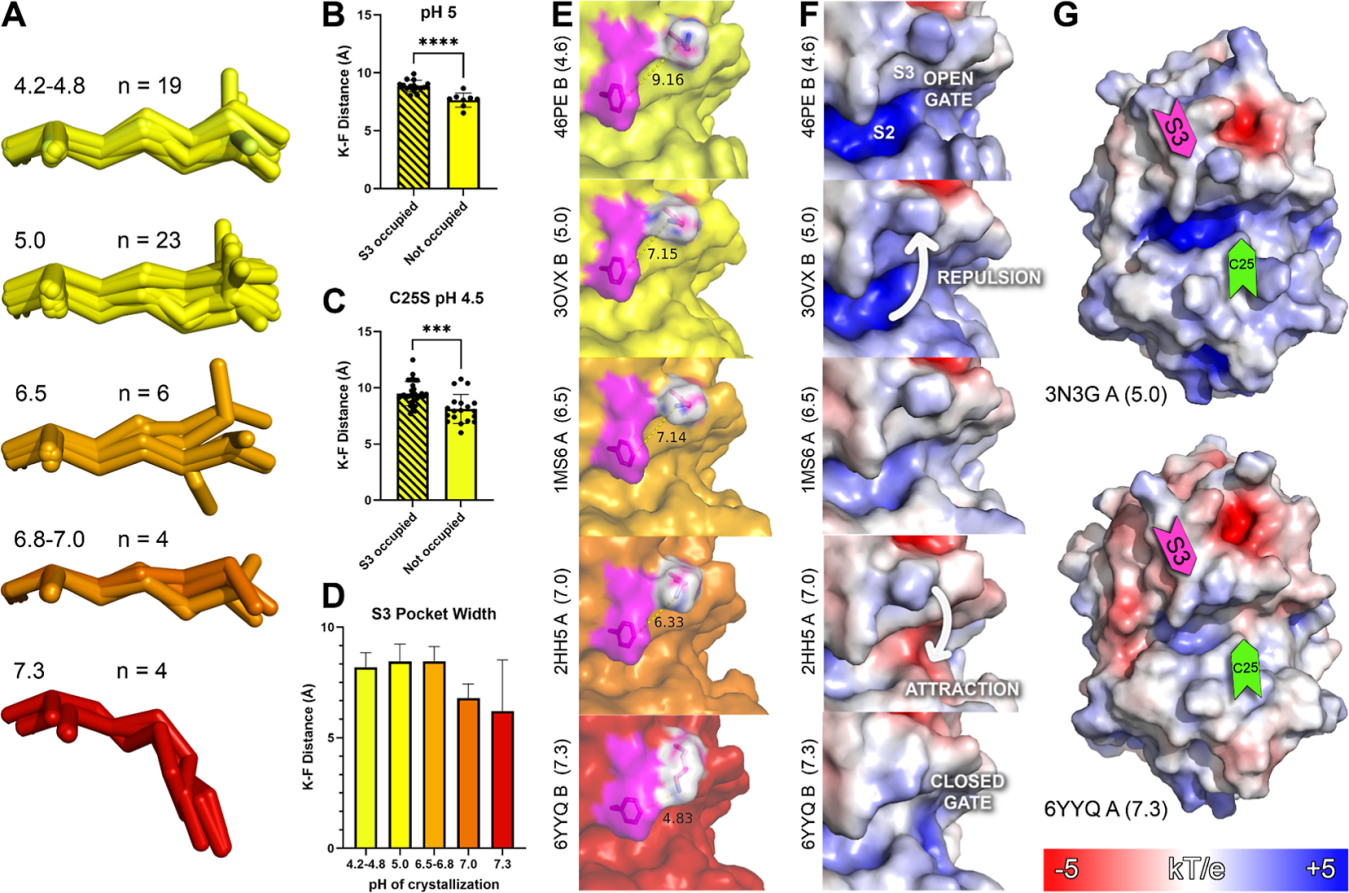
Active site conformation changes with pH. (A) Aligned Lys-64 residues for 56 WT cathepsin S structures* in the PDB, separated by pH. (B) S3 pocket width for all WT cathepsin S PDB structures at pH 5.0, comparing occupied and unoccupied pockets (*p* < 0.0001). (C) S3 pocket width for mutant C25S cathepsin S PDB structures at pH 4.5, comparing occupied and unoccupied pockets (*p* = 0.0002). (D) S3 pocket width of WT cathepsin S structures vs pH. (E) Example structures of the S3 pocket of cathepsin S closing as pH increases. (F) APBS (v3.4.1) electrostatic surface model for the same example S3 pockets. (G) APBS electrostatic surface models for PDB structures 3N3G chain A, calculated at its original pH of 5.0, and 6YYQ chain A, calculated at its original pH of 7.3. *PDB 9GJ2 chain B excluded due to alignment failure.

The S3 pocket of cathepsin S is unique across cysteine cathepsins (Supporting Information—Sequence Alignment). It is the main structural determinant allowing for the selectivity of cathepsin S-specific inhibitors, which are often designed around the nucleophilic amine of Lys-64.^103,116^ Out of the 57 structures of cathepsin S in the PDB that met our inclusion criteria, 34 had inhibitors that occupied this pocket (60%) (Supporting Information, S3 Pocket Width Measurements). Cathepsin S was initially described as challenging to crystallize in part because of this region of the protein: its first published structure was of a crystal that took 18 months to grow and did not include the loop region from residues 61 to 64. That loop section is considered to be intrinsically disordered, and its structure was only identified after being crystallized with an inhibitor, which held open the S3 pocket (it has since been successfully resolved in complex with many inhibitors that do not occupy S3).^109,110,116^ We confirmed that the size of the S3 pocket can flex to accommodate inhibitors by measuring WT cathepsin S structures that were resolved in this area and found that the width of the S3 pocket was significantly larger when occupied by an inhibitor (Figure 3B). This trend also held for C25S mutant structures, where the catalytic cysteine has been mutated to serine (Figure 3C).

By measuring the S3 pocket width of all WT cathepsin S structures, we observed that the S3 pocket size decreases with an increase in pH (Figure 3D,E). In a linear regression, both experimental pH (*p* = 0.0227) and presence of an inhibitor (*p* = 0.0078) were statistically significant (see Supporting Information—Multiple Linear Regression). This outcome is also shown clearly when comparing the available structures. Lys-64 descends into the active site starting at pH 7.0 and occupies the S3 pocket at pH 7.3, acting as a pH-dependent gate.

When the electrostatic potential of the surface of cathepsin S across pH levels was analyzed using the adaptive Poisson–Boltzmann solver (APBS), the S2 pocket was shown to be very positively charged below a pH of 7.0. The behavior of Lys-64 can be explained by the positively charged S2 pocket repelling the positively charged amino group of Lys-64 and causing it to be oriented away at lower pH. The S2 pocket becomes negatively charged only at a pH of 7.0, which explains why the positively charged amine of the flexible Lys-64 then descends, after which the S2 pocket becomes positively charged once again (Figure 3F).

The flip of Lys-64’s into the S3 pocket explains the pH-dependent specificity switch seen in our digest experiments. Peptide HRVK, which has a positively charged arginine in the P3 position, is not digested as rapidly above pH 7.0, while peptide LIFQ, which has the smaller uncharged amino acid isoleucine in P3, continues to be digested. If Lys-64 descends into the active site above pH 7, its positively charged amine would repel positively charged amino acids in the P3 position of substrates, like in peptide HRVK. Therefore, substrates with positively charged residues in P3 would be selected against in cathepsin S digests, which occur at pH above 7.

The digestion of peptide LIFE at pH 7.4 seen in the original screening digest may be explained by an electrostatic attraction between the negatively charged glutamic acid and the positively charged amino group in its “closed gate” orientation. The electrostatic surfaces generated by APBS show several other charge flips in the active site, which may contribute to the complex relationship between pH and specificity seen in our initial screening digests (Figure 3G).

## Discussion

The results from our digestion experiments suggest that cathepsin S can catalyze substrates with hydrophobic residues in the P3 position over a wider pH range than substrates with basic residues in the P3 position. This property would make the pH-dependent specificity switch of cathepsin S similar to that of cruzain, which also accepts hydrophobic residues at a wider pH range than basic residues, although their mechanisms are completely different.^97^ The width of the pH curves of cysteine proteases have previously been assumed to be controlled by the p*K*_a_ values of the Cys–His proton donor–acceptor pair,^111,112^ and this width for cathepsin S has previously been measured at 3.3 with a peak at pH 6.5, based on catalysis of the substrate Z-VVR|-MCA.^77,113^ For the substrate HRVK in Figure 2A, however, the curve width is estimated to be around 2. The available crystal structures of cathepsin S were therefore compared to identify possible causes, and a flip of Lys-64 into the S3 region of the active site was identified as the probable cause of this switch, although due to the limited number of PDB structures crystallized at pH > 6, further structures should be determined at higher pH levels to confirm this trend. The residues which make up the S3 pocket are specific to cathepsin S, and the pocket has been used as a specificity-determining region for inhibitors;^103,114,115^ determining the flexibility of this region in and outside of the lysosome may be critical for developing any inhibitor that is specific to both cathepsin S and any target biological compartment.

Many of the diseases associated with cathepsin S also involve drops in pH, and the pH-dependent specificity switch we observed can explain how cathepsin S contributes to pathological protein degradation in those conditions: as serum pH drops below 7.4, cathepsin S may switch from its controlled cell signaling mode to its destructive waste management mode. (For examples of cathepsin S-associated processes that are also associated with pH transitions, see Table 2.) Further investigations will reveal whether cathepsin S is the missing connection that explains the relationship between these environmental properties and the healthy functioning of a process or the severity of a disease or whether, in the complex communication relays of these biological systems, cathepsin S is only noise.

**Table 2:** pH Transitions in Healthy and Dysregulated Processes Known to Involve Cathepsin S

compartment or process	pH transition	cathepsin S associations
endolysosomal pathway	extracellular pH: 7.4	• degradation of antigens^2,79^
	endosomal pH: 7.4–6.5	• release of the invariant chain from MHC-II^3,49,50^
	lysosomal pH: 6.5–4.0 ^116^	
macrophage pericellular environment	pericellular pH when adhered to elastin: ∼6.0 ^117,118^	• extracellular matrix degradation after secretion by macrophages^12^
ischemic events	minimum pH: 6.3 ^119^	• contribution to reperfusion injury through degradation of structural proteins^10,45,52^
	lower pH is associated with more structural damage	• increased levels before and after ischemic events^44,120^
		• higher levels associated with more damage
		• may be protective in its role in revascularization during healing^47^
atherosclerotic plaques	varies from 6.8 to 7.55 within a plaque^121^ extracellular acidification is pro-atherosclerotic^122^	• role in plaque formation, expansion, destabilization, and rupture^12,13,15,16,74^
metabolic acidemia	mild: serum pH < 7.35 ^123^, severe: serum pH < 7.2; acidemia below this point is associated with disrupted ion exchange^124^	• associated with chronic kidney disease as well as renal dysfunction in lupus, both of which can cause acidosis^29,74,125–127^
		• associated with aortal stiffening in chronic kidney disease^71,128,129^
tumor microenvironments	range: 5.6–7.0 ^130^	• promotes tumor expansion and neovascularization^36,40,63,131–133^
COPD	average pH of exhaled breath in healthy controls: 7.35–7.65 ^134–137^ in COPD: 6.87–7.29 ^134–136^	• associated with the degradation of lung epithelial cells in COPD^56,57^
Alzheimer’s disease	decreased post-mortem brain and cerebrospinal fluid pH in Alzheimer’s disease patients^138–140^	• found at increased levels in the post-mortem brains of patients with Alzheimer’s disease^64,65^

## Conclusion

Cathepsin S experiences a pH-dependent specificity switch around pH 7.2, rejecting substrates with basic residues at the P3 position. This is explained by a conformational change in the active site: Lys-64 descends into the S3 pocket of the enzyme above that pH and acts as a pH-dependent gate. The pH-dependent structure of the cathepsin S active site may enable the creation of inhibitors that target specific microenvironments based on pH. This pH-dependent specificity may also explain the dysregulated activity of cathepsin S in disease environments that feature extracellular pH drops, such as atherosclerotic plaques and tumors.

## Supplementary Material



## Notes

S3 pocket sequence of human cathepsin S aligned with homologous sequences; counts of wild-type (WT) cathepsin S structures available in the Protein Data Bank (PDB) by their pH of crystallization; counts of WT cathepsin S structures available in the Protein Data Bank (individual chains) by the presence and absence of an inhibitor structure in the S3 pocket of their active site; all individual chains of cathepsin S available in the PDB as of October 2024; multiple linear regression used to estimate the relationship between experimental pH, presence of inhibitor in the S3 pocket, and the S3 pocket width of wild-type cathepsin S structures available in the PDB; graph of S3 pocket width vs experimental pH colored by presence of an inhibitor in the S3 pocket; multiple linear regression model; parameter estimates; peptides selected for initial screening and their sources; experimental results showing the lack of impact that 2.4% isopropanol has on the titration curve of citrate-phosphate buffer; pH curve for peptide LVVRALPQGP compared to the pH curve for LIFQQGHPDH; substrate vs product area curve for the C terminal product of LVVRALPQGP hydrolysis by cathepsin S; and substrate vs product area curve for the N terminal product of LVVRALPQGP hydrolysis by cathepsin S (PDF)
