# Supplementary material for: The pH-Dependent Specificity of Cathepsin S and Its Implications for Inflammatory Communications and Disease
Source: Biochemistry. 2025 Jul 19;64(18):3841–53. doi: 10.1021/acs.biochem.5c00287 (PMC12444987; doi:10.1021/acs.biochem.5c00287)
Supplement: Supplementary file 1 [file bi5c00287_si_001.pdf]

SUPPLEMENTARY MATERIALS

The pH-dependent specificity of cathepsin S and its implications for inflammatory communications and disease

Authors

Riley DeHority[1], Laura I. Gil Pineda[2], Kari Cochran[1], Bentley Chen[1], Daniel Bratek[1], Richard F. Helm[2], Justin A. Lemkul[2], Chenming Zhang*[1]

[1] Virginia Polytechnic Institute and State University, Department of Biological Systems Engineering, Blacksburg, Virginia, 24061, USA
[2] Virginia Polytechnic Institute and State University, Department of Biochemistry, Blacksburg, Virginia, 24061, USA
*Corresponding author, chzhang2@vt.edu

Sequence Alignment

Table S1. The S3 pocket sequence of human cathepsin S, aligned with homologous sequences in the ten other human cysteine cathepsins. Lys-64 and its homologs, when present, are shown in bold.

Name	Sequence	Start	End	NCBI Sequence ID
Cathepsin S	GN**K**GCNGGF	62	70	NP_004070.3
Cathepsin L	GN**E**GCNGGL	119	127	NP_001244900.1
Cathepsin V	GN**Q**GCNGGF	174	182	NP_001188504.1
Cathepsin K	–N**D**GCGGGY	174	181	NP_000387.1
Cathepsin H	NN**H**GCQGGL	177	185	NP_004381.2
Cathepsin F	MD**K**ACMGGL	329	337	NP_003784.2
Cathepsin C	AQ**–**GCEGGF	295	302	NP_001805.4
Cathepsin O	NN**Y**GCNGG–	166	173	NP_001325.1
Cathepsin W	GD**–**GCHGGF	188	195	NP_001326.3
Cathepsin Z	GN**A**GSCEGG–	127	135	NP_001327.2
Cathepsin B	GD**–**GCNGGY	146	153	NP_001371643.1

S3 Pocket Width Measurements

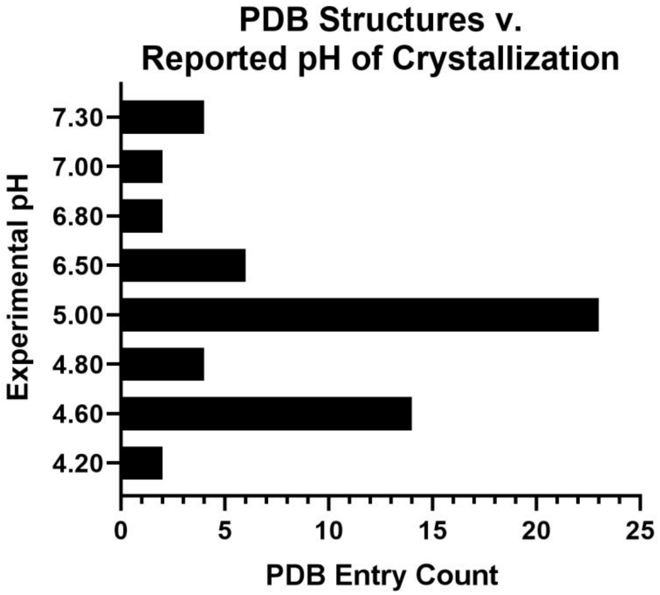

Figure S1: Counts of wild-type (WT) structures available in the Protein Data Bank (PDB) by their pH of crystallization.

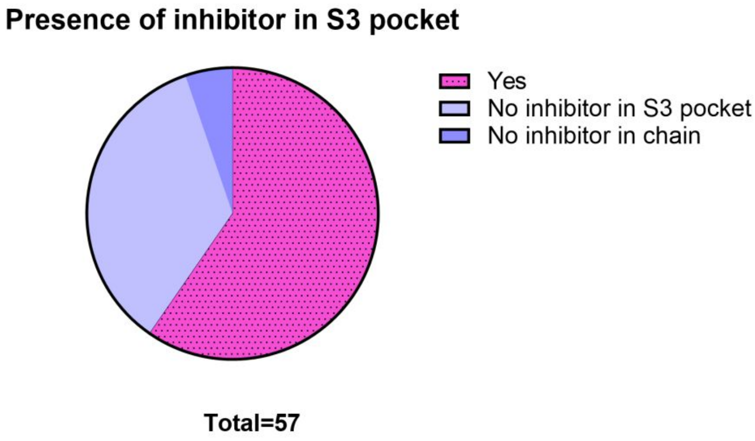

Figure S2: Counts of WT structures available in the Protein Data Bank (individual chains) by the presence and absence of an inhibitor structure in the S3 pocket of their active site. Three structures were co-crystallized with inhibitors but their chain structures did not contain them.

Table S2. All individual chains of cathepsin S available in the PDB as of October 2024. Ligand type description drawn from submission information.

PDB Code	Experimental pH	Author	Resolution (Å)	Ligand Type	Mutation	Chain	S3 occupied	K-F Distance (Å)
1GLO	4.1	Tuckenburg 2002	2.2	APO	C25S	A		
6YYO	4.2	Wagener 2021	1.5	Noncovalent - not in binding pocket	WT	A	No	6.86
6YYO	4.2	Wagener 2021	1.5	Noncovalent - not in binding pocket	WT	B	No	8.19
5QBZ	4.5	Bembenek 2017	2.8	Heterocyclic ligand	C25S	A	Yes	10.69
5QBV	4.5	Bembenek 2017	1.796	Heterocyclic ligand	C25S	A	Yes	9.31
5QBV	4.5	Bembenek 2017	1.796	Heterocyclic ligand	C25S	B	Yes	9.43
5QBW	4.5	Bembenek 2017	3.01	Heterocyclic ligand	C25S	A	Yes	10.67
5QBY	4.5	Bembenek 2017	2.25	Heterocyclic ligand	C25S	A	Yes	9.87
5QBY	4.5	Bembenek 2017	2.25	Heterocyclic ligand	C25S	B	Yes	11.20
5QC4	4.5	Bembenek 2017	2	Heterocyclic ligand	C25S	A	Yes	9.12
5QC4	4.5	Bembenek 2017	2	Heterocyclic ligand	C25S	B	Yes	8.72
5QCD	4.5	Bembenek 2017	1.95	Heterocyclic ligand	C25S	A	No	7.47
5QCF	4.5	Bembenek 2017	2.1	Heterocyclic ligand	C25S	A	No	6.99
5QCI	4.5	Bembenek 2017	2.179	Heterocyclic ligand	C25S	A	No	10.37
2FYE	4.5	Somoza 2006	2.2	Irreversible inhibitor	E59K, K104A, S111C, E193K	A		
2G6D	4.5	Somoza 2006	2.5	Inhibitor (other)	S111C, E193K	A		
5QBU	4.5	Bembenek 2017	2.78	Heterocyclic ligand	C25S	A	No	10.77

5QBU	4.5	Bembenek 2017	2.78	Heterocyclic ligand	C25S	B	No	8.63
5QBX	4.5	Bembenek 2017	2.1	Heterocyclic ligand	C25S	A	Yes	9.74
5QBX	4.5	Bembenek 2017	2.1	Heterocyclic ligand	C25S	B	Yes	9.48
5QC0	4.5	Bembenek 2017	1.9	Heterocyclic ligand	C25S	A	Yes	9.18
5QC0	4.5	Bembenek 2017	1.9	Heterocyclic ligand	C25S	B	Yes	8.18
5QC1	4.5	Bembenek 2017	2.082	Heterocyclic ligand	C25S	A	Yes	9.36
5QC1	4.5	Bembenek 2017	2.082	Heterocyclic ligand	C25S	B	Yes	9.25
5QC2	4.5	Bembenek 2017	2.26	Heterocyclic ligand	C25S	A	Yes	9.22
5QC2	4.5	Bembenek 2017	2.26	Heterocyclic ligand	C25S	B	Yes	10.19
5QC3	4.5	Bembenek 2017	1.98	Heterocyclic ligand	C25S	A	Yes	8.40
5QC3	4.5	Bembenek 2017	1.98	Heterocyclic ligand	C25S	B	Yes	8.37
5QC5	4.5	Bembenek 2017	2.4	Heterocyclic ligand	C25S	A	Yes	9.30
5QC5	4.5	Bembenek 2017	2.4	Heterocyclic ligand	C25S	B	Yes	9.24
5QC6	4.5	Bembenek 2017	2.1	Heterocyclic ligand	C25S	A	Yes	10.70
5QC6	4.5	Bembenek 2017	2.1	Heterocyclic ligand	C25S	B	Yes	10.67
5QC8	4.5	Bembenek 2017	1.74	Heterocyclic ligand	C25S	A	Yes	7.98
5QC8	4.5	Bembenek 2017	1.74	Heterocyclic ligand	C25S	B	Yes	12.48
5QC9	4.5	Bembenek 2017	2	Heterocyclic ligand	C25S	A	Yes	8.84
5QC9	4.5	Bembenek 2017	2	Heterocyclic ligand	C25S	B	Yes	7.93
5QCB	4.5	Bembenek 2017	2.2	Heterocyclic ligand	C25S	A	Yes	9.27

5QCB	4.5	Bembenek 2017	2.2	Heterocyclic ligand	C25S	B	Yes	9.05
5QCC	4.5	Bembenek 2017	1.8	Heterocyclic ligand	C25S	A	No	8.63
5QCC	4.5	Bembenek 2017	1.8	Heterocyclic ligand	C25S	B	No	8.74
5QCE	4.5	Bembenek 2017	2.78	Heterocyclic ligand	C25S	A	Yes	10.79
5QCE	4.5	Bembenek 2017	2.78	Heterocyclic ligand	C25S	B	No	10.39
5QCG	4.5	Bembenek 2017	2.7	Heterocyclic ligand	C25S	A	No	8.32
5QCG	4.5	Bembenek 2017	2.7	Heterocyclic ligand	C25S	B	No	7.93
5QCA	4.5	Bembenek 2017	2.29	Heterocyclic ligand	C25S	A	No	8.19
5QCA	4.5	Bembenek 2017	2.29	Heterocyclic ligand	C25S	B	No	7.94
5QCH	4.5	Bembenek 2017	2.2	Heterocyclic ligand	C25S	A	No	7.99
5QCH	4.5	Bembenek 2017	2.2	Heterocyclic ligand	C25S	B	No	7.55
5QCH	4.5	Bembenek 2017	2.2	Heterocyclic ligand	C25S	C	No	7.05
5QCH	4.5	Bembenek 2017	2.2	Heterocyclic ligand	C25S	D	No	7.41
5QCJ	4.5	Bembenek 2017	2	Heterocyclic ligand	C25S	A	No	6.80
5QCJ	4.5	Bembenek 2017	2	Heterocyclic ligand	C25S	B	No	6.00
5QCJ	4.5	Bembenek 2017	2	Heterocyclic ligand	C25S	C	No	6.97
5QC7	4.5	Bembenek 2017	1.9	Heterocyclic ligand	C25S	A	Yes	7.70
5QC7	4.5	Bembenek 2017	1.9	Heterocyclic ligand	C25S	B	Yes	9.44
3IEJ	4.56	Bembenek 2009	2.18	Inhibitor (other)	C25S	A	No	
3IEJ	4.56	Bembenek 2009	2.18	Inhibitor (other)	C25S	B	No	
2R9M	4.6	Ward 2007	1.97	Ligand (other)	WT	A	Yes	8.26

2R9M	4.6	Ward 2007	1.97	Ligand (other)	WT	B	Yes	8.78
2R9O	4.6	Ward 2007	2	Ligand (other)	WT	A	Yes	8.41
2R9O	4.6	Ward 2007	2	Ligand (other)	WT	B	Yes	8.9
4P6E	4.6	Wang 2014	1.8	Inhibitor (other)	WT	A	Yes	8.55
4P6E	4.6	Wang 2014	1.8	Inhibitor (other)	WT	B	Yes	9.16
2R9N	4.6	Ward 2007	2	Ligand (other)	WT	A	Yes	8.10
2R9N	4.6	Ward 2007	2	Ligand (other)	WT	B	Yes	8.92
4P6G	4.6	Wang 2014	1.58	Inhibitor (other)	WT	A	Yes	7.56
4P6G	4.6	Wang 2014	1.58	Inhibitor (other)	WT	B	Yes	7.50
4P6G	4.6	Wang 2014	1.58	Inhibitor (other)	WT	C	Yes	7.59
4P6G	4.6	Wang 2014	1.58	Inhibitor (other)	WT	D	Yes	7.75
9GJ2	4.6	Falke 2024	1.15	Ketoamide	WT	A	No	9.22
9GJ2	4.6	Falke 2024	1.15	Ketoamide	WT	B	No	7.94
6YYN	4.8	Wagener 2020	2.22	Noncovalent - not in binding pocket	WT	A	No	8.17
6YYN	4.8	Wagener 2020	2.22	Noncovalent - not in binding pocket	WT	B	No	7.48
6YYR	4.8	Wagener 2020	1.3	Noncovalent - not in binding pocket	WT	A	No	F not resolved
6YYR	4.8	Wagener 2020	1.3	Noncovalent - not in binding pocket	WT	B	No	F not resolved
2H7J	5	Patterson 2006	1.5	Nonpeptidic inhibitor	WT	A	Yes	9.06
2H7J	5	Patterson 2006	1.5	Nonpeptidic inhibitor	WT	B	Yes	9.46
2HXZ	5	Patterson 2006	1.9	Nonpeptidic inhibitor	WT	A	Yes	9.40

2HXZ	5	Patterson 2006	1.9	Nonpeptidic inhibitor	WT	B	Yes	8.72
2HXZ	5	Patterson 2006	1.9	Nonpeptidic inhibitor	WT	C	Yes	9.91
3OVX	5	Fradera 2010	1.49	Covalent inhibitor	WT	A	No	7.65
3OVX	5	Fradera 2010	1.49	Covalent inhibitor	WT	B	No	7.15
2G7Y	5	Somoza, 2006	2	Inhibitor (other)	WT	A	Yes	8.09
2G7Y	5	Somoza, 2006	2	Inhibitor (other)	WT	B	Yes	8.69
3N4C	5	Fradera 2010	1.9	Nitrile inhibitor	WT	A	No	6.54
3N4C	5	Fradera 2010	1.9	Nitrile inhibitor	WT	B	No	7.75
2FQ9	5	Somoza 2006	1.65	Nitrile inhibitor	WT	A	Yes	8.93
2FQ9	5	Somoza 2006	1.65	Nitrile inhibitor	WT	B	Yes	8.67
2FRA	5	Somoza 2006	1.9	Nitrile inhibitor	WT	A	Yes	9.02
2FRA	5	Somoza 2006	1.9	Nitrile inhibitor	WT	B	Yes	8.92
2FT2	5	Somoza 2006	1.7	Inhibitor (other)	WT	A	Yes	8.84
2FT2	5	Somoza 2006	1.7	Inhibitor (other)	WT	B	Yes	8.46
3N3G	5	Fradera 2010	1.6	Nitrile inhibitor	WT	A	No	8.67
3N3G	5	Fradera 2010	1.6	Nitrile inhibitor	WT	B	No	7.93
2FRQ	5	Somoza 2006	1.6	Inhibitor (other)	WT	A	No	7.83
2FRQ	5	Somoza 2006	1.6	Inhibitor (other)	WT	B	No	7.73
2FUD	5	Somoza 2006	1.95	Inhibitor (other)	WT	A	Yes	8.75
2FUD	5	Somoza 2006	1.95	Inhibitor (other)	WT	B	Yes	8.05
1NPZ	5.5	Pauly 2003	2	Inhibitor (other)	S47T	A	Yes	
1NPZ	5.5	Pauly 2003	2	Inhibitor (other)	S47T	B	Yes	

1NQC	5.5	Pauly 2003	1.8	Inhibitor (other)	S47T	A	Yes	
1MS6	6.5	Ward 2002	1.9	Nitrile inhibitor	WT	A	Yes	7.14
2F1G	6.5	Spraggon 2006	1.9	Inhibitor (other)	WT	A	Yes	8.33
2F1G	6.5	Spraggon 2006	1.9	Inhibitor (other)	WT	B	Yes	9.19
2HHN	6.5	Spraggon 2006	1.55	Noncovalent - not in binding pocket	WT	A	No (no inhibitor)	8.21
2HHN	6.5	Spraggon 2006	1.55	Noncovalent - not in binding pocket	WT	B	Yes	8.13
8RND	6.5	Petruzella 2024	1.56	Peptide based inhibitor	WT	A	Yes	9.11
6YYP	6.8	Wagener 2020	2.05	Noncovalent - not in binding pocket	WT	A	No	8.75
6YYP	6.8	Wagener 2020	2.05	Noncovalent - not in binding pocket	WT	B	No (no inhibitor)	8.8
2HH5	7	Spraggon 2006	1.8	Inhibitor (other)	WT	A	Yes	6.33
2HH5	7	Spraggon 2006	1.8	Inhibitor (other)	WT	B	Yes	7.24
8PI3	7	Petruzella 2023	1.73	Peptide based inhibitor	Y132D	A		
6YYQ	7.3	Wagener 2020	2.51	Noncovalent - not in binding pocket	WT	A	No	5.15
6YYQ	7.3	Wagener 2020	2.51	Noncovalent - not in binding pocket	WT	B	No	4.83
6YYQ	7.3	Wagener 2020	2.51	Noncovalent - not in binding pocket	WT	C	No	5.14

6YYQ	7.3	Wagener 2020	2.51	Noncovalent - not in binding pocket	WT	D	No (no inhibitor)	9.67
2C0Y	7.5	Kaulmann 2005	2.1	APO	C25A	A		

Multiple Linear Regression

A multiple linear regression (GraphPad Prism 10.3.1) was used to estimate the relationship between experimental pH, presence of inhibitor in the S3 pocket, and the S3 pocket width (measuring from the terminal amine of Lys-64 to the ζ carbon of Phe-70) of wild-type cathepsin S structures available in the PDB. Two of the WT structures (6YYR chains A and B) were not used, as their S3 pocket structure was not resolved. An initial analysis showed that the interaction effect between the independent variables was not significant ($p = 0.9762$) therefore it was excluded from the model.

Equation. Multiple linear regression. Presence of inhibitor is a binary variable, with Yes = 1 and No = 0.

$$S3 \text{ Pocket Width } = 9.457 - 0.3343 * \text{Experimental pH } + 0.7482 * \text{Presence of inhibitor in } S3$$

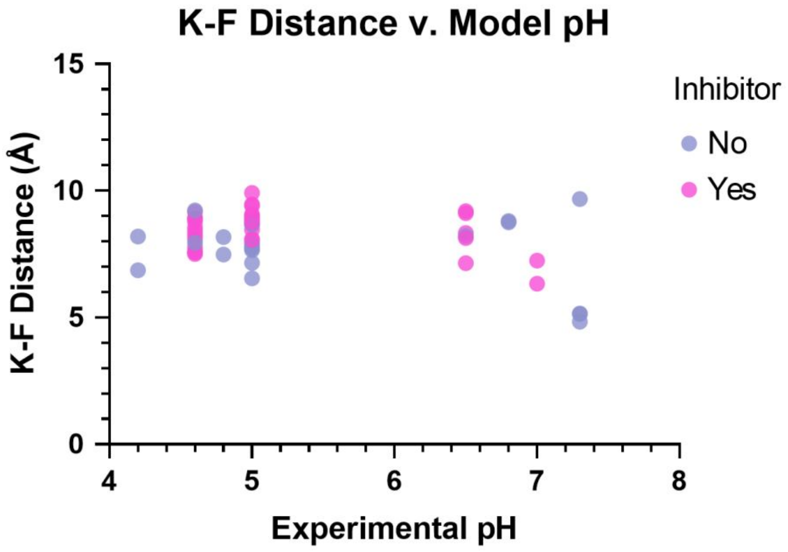

Figure S3. Graph of S3 pocket width vs. experimental pH colored by presence of an inhibitor in the S3 pocket.

Table S3. Multiple linear regression model.

**Analysis of Variance**	**Sum of Squares**	**Degrees of Freedom**	**Mean Square**	**F (DFn, DFd)**	**P value**
Regression	14.76	2	7.382	F (2, 52) = 8.071	P=0.0009
Experimental pH	5.042	1	5.042	F (1, 52) = 5.513	P=0.0227
Inhibitor	7.019	1	7.019	F (1, 52) = 7.674	P=0.0078
Residual	47.56	52	0.9146		
Total	62.33	54			

Table S4. Parameter estimates.

**Parameter estimates**	**Variable**	**Estimate**	**Standard error**	**95% Confidence Interval (asymptotic)**	**|t|**	**P value**
β0	Intercept	9.457	0.8169	7.818 to 11.10	11.58	<0.0001
β1	Experimental pH	-0.3343	0.1424	-0.6200 to -0.04860	2.348	0.0227
β2	Inhibitor[Yes]	0.7482	0.2701	0.2062 to 1.290	2.770	0.0078

Peptide selection

Table S5. Peptides selected for initial screening and their sources.

Source	Source Molecule	Sequence	pH of Discovery	Reported MW (LifeTein)
2020 Panwar	Elastin (Assembled from mass sequencing results)	IGPGGVAAAA	5.5	782.89
2006 Rückrich	Invariant Chain	LPMGALPQGP	5.5	980.178
2006 Choe & 2006 Rückrich	Recombinant library & invariant chain	HRVKALPQGP	5.5	1102.3
2011 Biniossek	HEK 293 cell lysates (Assembled from mass sequencing results)	LIFQQGHPDH	6, 7.5	1191.32
2011 Biniossek	HEK 293 cell lysates (Assembled from mass sequencing results)	LIFEQGHPDH	6, 7.5	1192.3
Review	Least accepted residues across studies	LRDRPRMMRR	N/A	1385.7

Buffers

Citrate-phosphate buffer was used for pH curve digests, as it has a stable titration curve over the entire pH range of cathepsin S activity. 0.3 M citric acid was titrated into 0.1 M Na_2HPO_4, ensuring that across the range, the buffer would be at or near 300 mOsM. To reduce the potential pKa impact of the isopropyl alcohol used to solubilize the peptide substrates, a weak solution of isopropanol was identified (2.4% v/v) which could dissolve all of the selected peptides. The impact of this concentration of isopropanol on the titration curve of citrate-phosphate buffer was tested by titrating the buffer in double distilled water, versus 2.4% isopropanol.

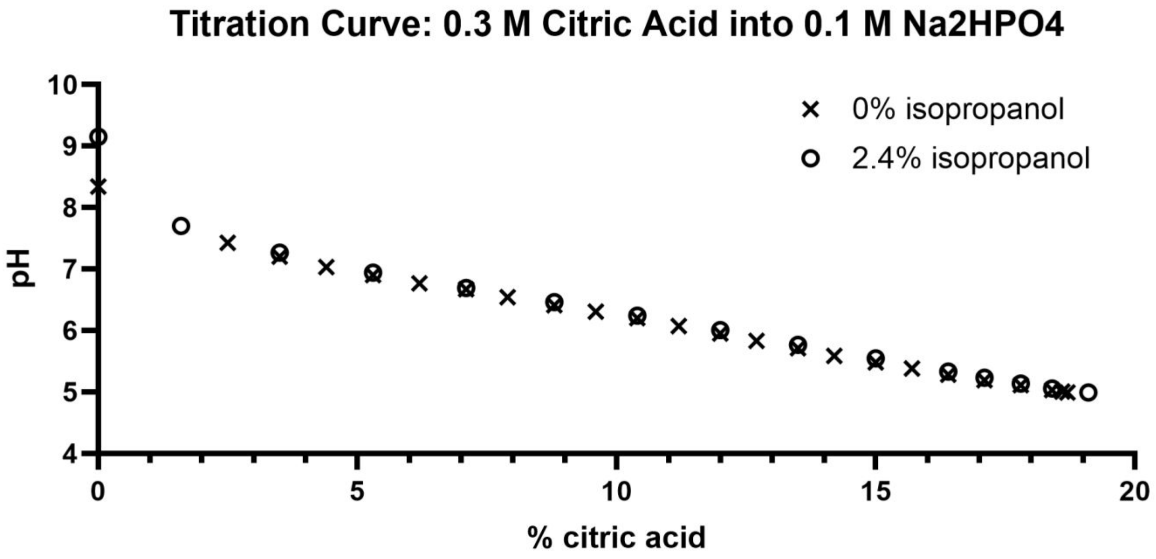

Figure S4. Experimental results showing the lack of impact that 2.4% isopropanol has on the titration curve of citrate-phosphate buffer.

LVVR pH Curve

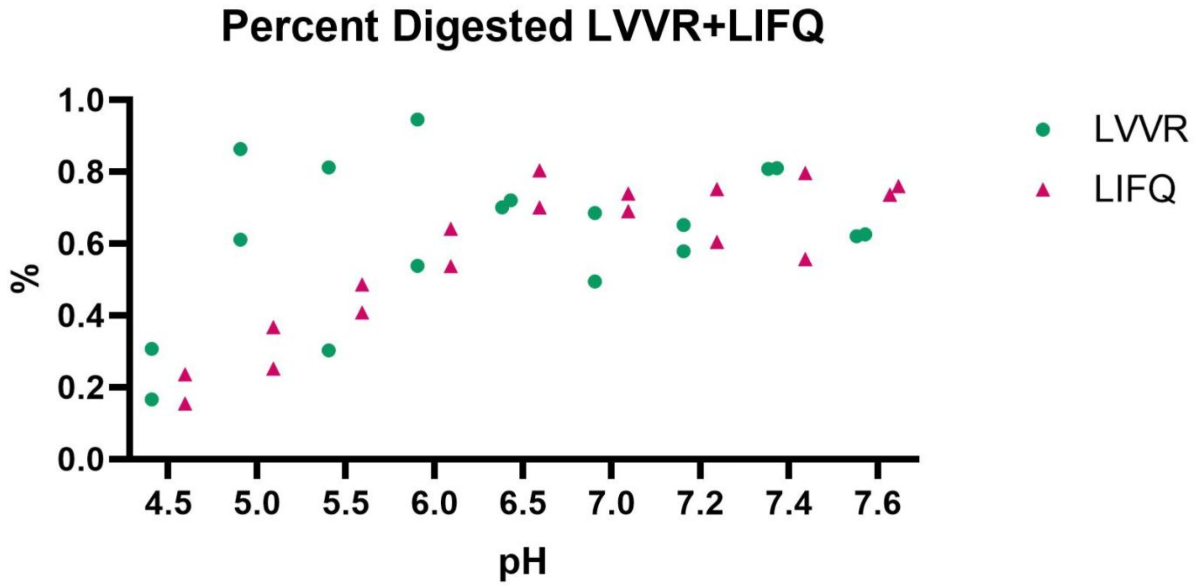

Figure S5. pH curve for peptide LVVRALPQGP compared to the pH curve for LIFQQGHPDH (n=2).

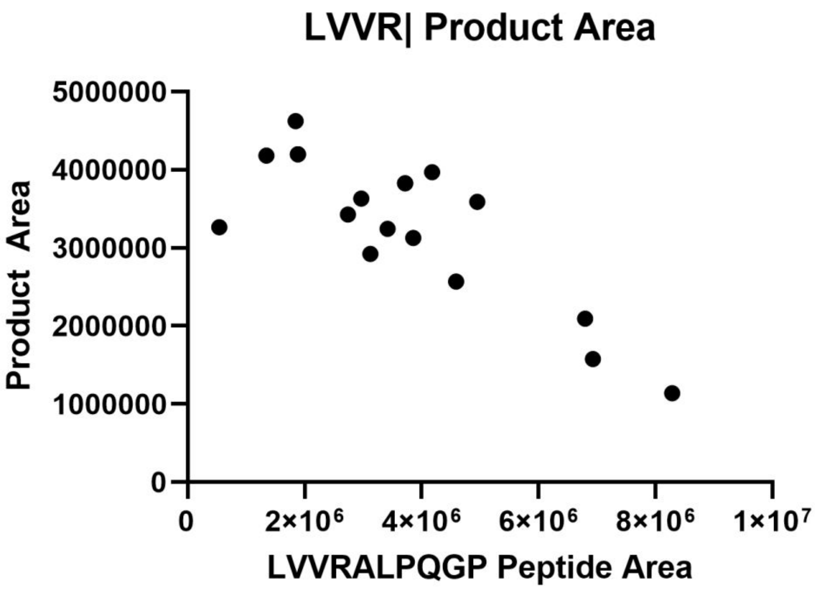

Figure S6. Substrate v. Product area curve for the C terminal product of LVVRALPQGP hydrolysis by cathepsin S.

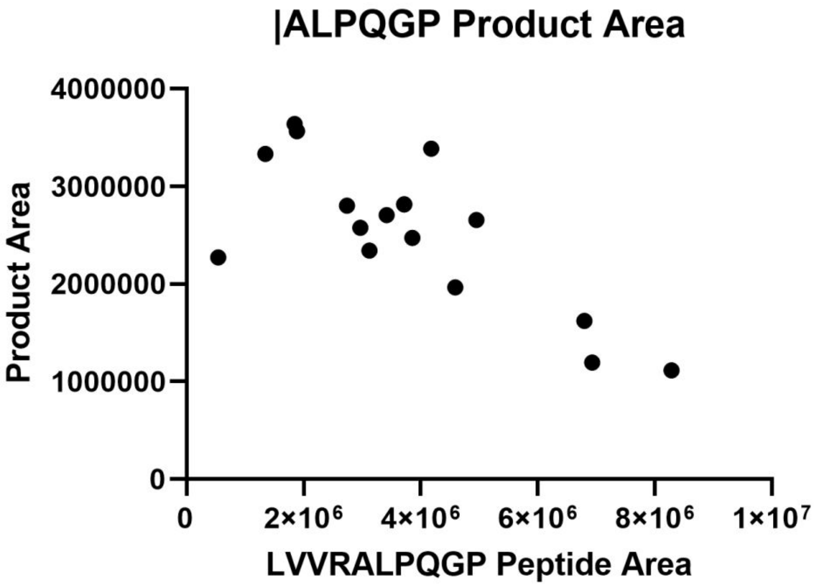

Figure S7. Substrate v. Product area curve for the N terminal product of LVVRALPQGP hydrolysis by cathepsin S.
